# Research progresses and hotspots on glucose metabolic reprogramming in breast cancer: a bibliometric analysis over the past two decades

**DOI:** 10.3389/fonc.2024.1493996

**Published:** 2025-01-14

**Authors:** Lei Huang, Wenyue Zhao, Lamei Sun, Dong Niu, Xiaodan Zhu, Chunhui Jin

**Affiliations:** Department of Oncology, Wuxi Affiliated Hospital of Nanjing University of Chinese Medicine, Wuxi, China

**Keywords:** bibliometrics, breast cancer, glucose metabolism reprogramming, tumor microenvironment, antimetabolic therapy

## Abstract

**Background:**

Abnormal energy metabolism is a prominent characteristic of cancers. Increasing evidence has suggested the involvement of glucose metabolism reprogramming in the progression of breast cancer (BC). This article aims to provide a comprehensive overview of glucose metabolism reprogramming in BC through a bibliometric analysis.

**Methods:**

Relevant literatures published from 2004 to 2024 were searched in the Web of Science Core Collection database, and a bibliometric analysis was conducted using VOSviewer, CiteSpace, and Bibliometrix.

**Results:**

In total, 957 publications reporting glucose metabolism reprogramming in BC were included, showing an increasing trend in the annual publication outputs. China ranked first in publication outputs, and the United States of America (USA) had a dominant place in citation counts. The research achievements of Thomas Jefferson University in the USA were at the forefront and widely cited. Lisanti, Michael P., and Sotgia, Federica were the most productive authors. Keyword analysis suggested that the mechanisms of glucose metabolism reprogramming in BC and related therapeutic strategies were the research hotspots.

**Conclusion:**

This study, for the first time, elucidated the progresses and hotspots of in the research on glucose metabolism reprogramming in BC, highlighting its potential role in treating BC. Considering that the glycolytic reprogramming of BC is a complex biological process, it is imperative for countries to enhance cooperation in the pursuit of effective antimetabolic therapies to overcome challenges in BC treatment.

## 1 Introduction

Breast cancer (BC), with an incidence and a mortality just next to those of lung cancer worldwide, seriously threatens the health of women (1). Owing to advances in molecular biology, multiple therapeutic strategies are available to BC patients, although recurrence, metastasis, and resistance greatly challenge their therapeutic outcomes (2).

In recent years, intracellular and extracellular energy metabolism, especially glucose metabolism reprogramming, have been highlighted in mediating the phenotypes of BC cells (3–5). The Warburg effect describes that tumor cells produce energy via glycolysis for unlimited proliferation (6, 7). Researchers have found that the Warburg effect involves numerous tumor biological processes, such as gene transcription, cell cycle, and immune evasion (8–10). Currently, one research hotspot is the glucose metabolism of tumor cells mediated by the Warburg effect, and measures targeting this mechanism have emerged to reshape the landscape of cancer therapy.

A bibliometric analysis can quantitate the productivity and impact of a particular research field by statistically analyzing relevant data of publications. Through tracking the relationships and developmental trends among knowledge clusters, a bibliometric analysis is expected to guide future research (11). This research method plays a crucial role in the medical field by systematically evaluating basic experiments, clinical studies, and authoritative guidelines, thereby providing both a theoretical foundation and a practical basis for the effective translation of medical knowledge into clinical practice (12). So far, there lacks a comprehensive overview of the progresses and hotspots in the research on glucose metabolism reprogramming in BC.

Through a deep mining of publication outputs, collaboration network, journal metrics, and citation and keyword analyses, the present study identified strengths and gaps in previous research, as well as provided valuable inspirations into future research on glucose metabolism reprogramming in BC or other types of cancers.

## 2 Methods

### 2.1 Data source and publication retrieval

**Figure 1:**
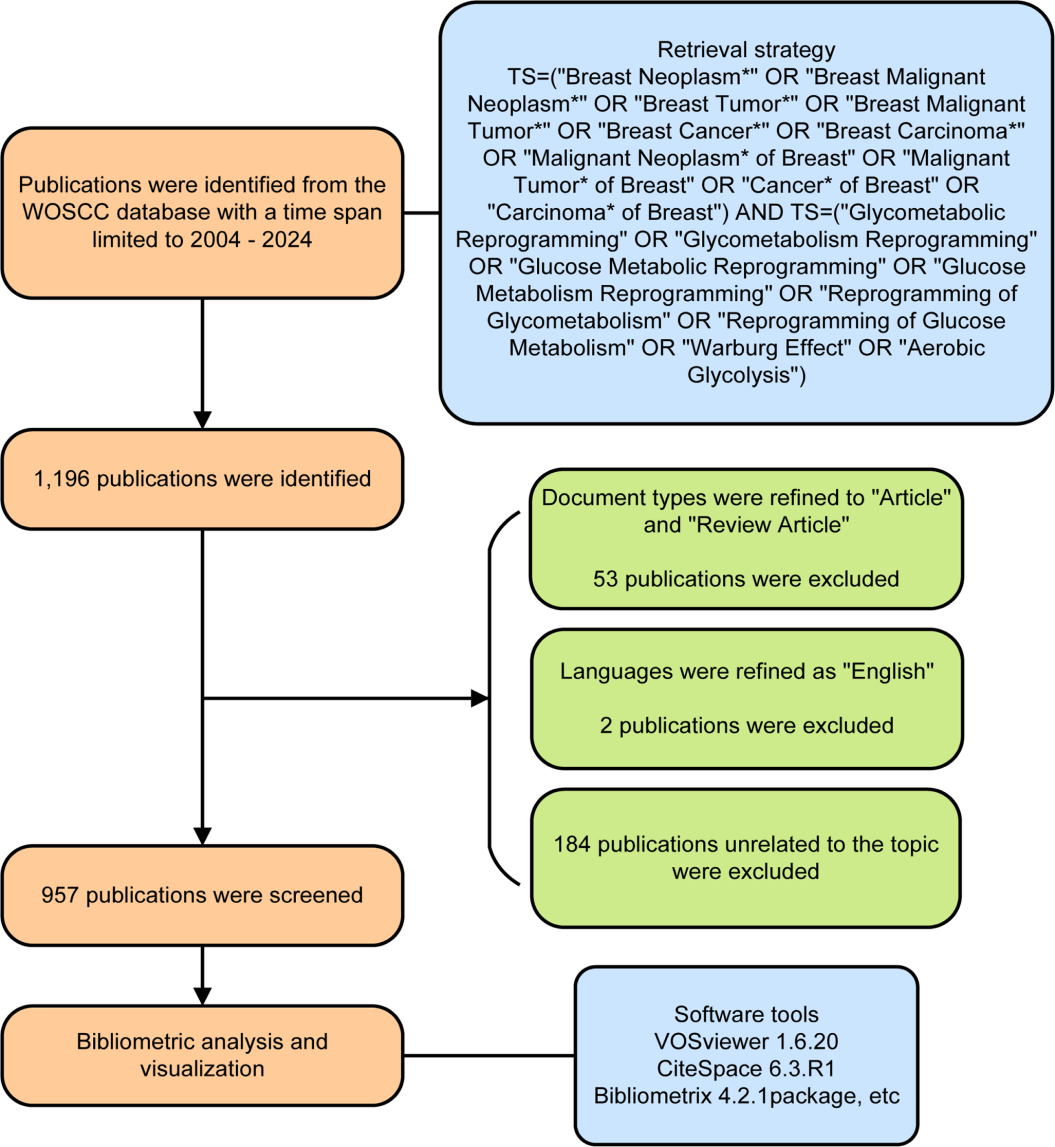
A flow chart of publication retrieval.

Data collection was performed using the Web of Science (WOS), which is an established platform to track, evaluate and compare scientific research (13). Briefly, literature was searched on May 12, 2024 in the Web of Science Core Collection (WOSCC) database by querying TS=(“Breast Neoplasm*” OR “Breast Malignant Neoplasm*” OR “Breast Tumor*” OR “Breast Malignant Tumor*” OR “Breast Cancer*” OR “Breast Carcinoma*” OR “Malignant Neoplasm* of Breast” OR “Malignant Tumor* of Breast” OR “Cancer* of Breast” OR “Carcinoma* of Breast”) AND TS=(“Glycometabolic Reprogramming” OR “Glycometabolism Reprogramming” OR “Glucose Metabolic Reprogramming” OR “Glucose Metabolism Reprogramming” OR “Reprogramming of Glycometabolism” OR “Reprogramming of Glucose Metabolism” OR “Warburg Effect” OR “Aerobic Glycolysis”), with a time period spanning from 2004 to 2024. A total of 1,196 publications were initially retrieved. After excluding 53 non-articles or non-review articles, 2 publications in non-English language, and 184 with unrelated topics, 957 eligible publications were finally involved in our study. A flow chart of publication retrieval is depicted in **Figure 1**. The full-texts and citations were exported and saved in the file format of plain text for further research.

### 2.2 Software tools for bibliometrics

The VOSviewer 1.6.20, CiteSpace 6.3.R1, and the Bibliometrix 4.2.1 package in R language (https://www.bibliometrix.org) were employed for bibliometric analysis of data collected from the WOSCC database. Briefly, the VOSviewer was used to visualize a bibliometric network through analyzing countries (regions), institutions, authors, journals, citations, keywords and highly cited publications; the CiteSpace to visualize a dual-map overlay of journals and keyword bursts; the Bibliometrix to generate a three-field plot among authors, countries (regions), and journals; Microsoft Office Excel 2019 to plot the trend of annual publication outputs to grasp publication dynamics; the Scimago Graphica 1.0.42 to construct a publication and cooperation network between countries (regions); Pajek 5.18 was used to adjust the layout structure of the clustering to optimize the visualized networks.

## 3 Results

### 3.1 Global publications

**Figure 2:**
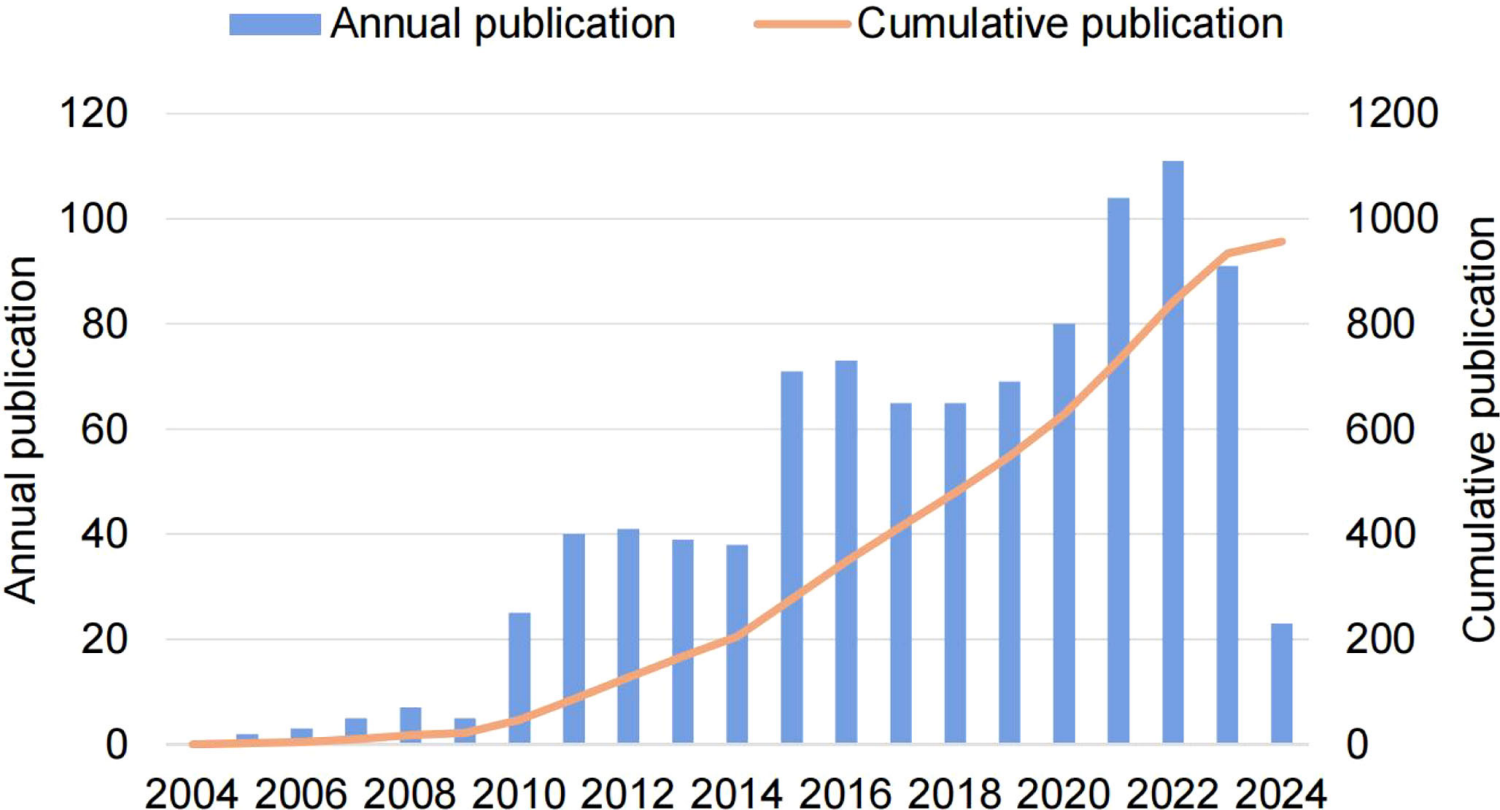
The trend of global publications of glucose metabolism reprogramming in BC from 2004 to 2024.

A total of 957 eligible publications were included, involving 692 (72.3%) original articles and 265 (27.7%) reviews. Before 2010, the annual number of publications did not exceed 10. With the rapid development of medical technologies like mass spectrometry and metabolomics, the annual number of publications gradually increased after 2010, but the growth rate fluctuated. Notably, a significant turning point occurred in 2015, with a sharp increase in annual output by three times that of 2010. By 2021, the annual number of publications exceeded 100, showing an overall increased trend with a slight fluctuation. After fitting the data, a significant relationship emerged between the year of publication and the cumulative annual number of publications (R2 = 0.996, **Figure 2**), suggesting the mounting enthusiasm on glucose metabolism reprogramming in the research of BC.

### 3.2 Distribution of countries (regions)

**Figure 3:**
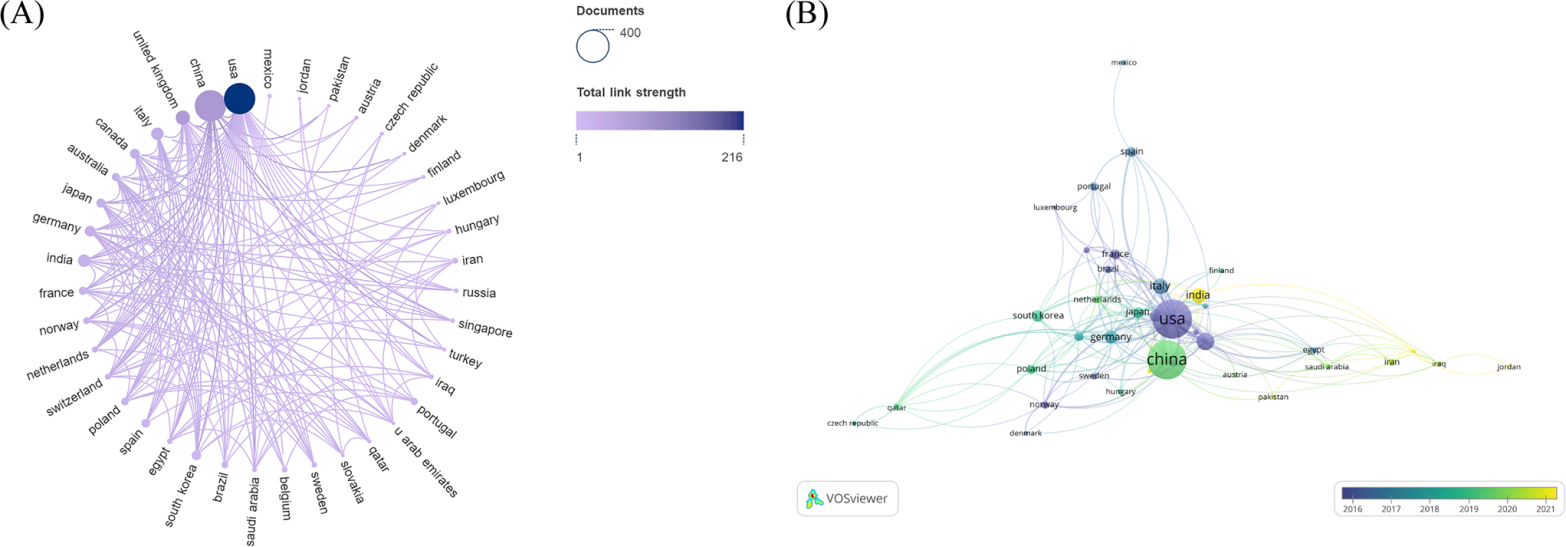
Country/region distribution of publications on glucose metabolism reprogramming in BC. **(A)** A network of countries/regions contributing to publication outputs, and their corporation. The sizes of nodes reflect the number of publications by countries (regions), and the thicknesses of the lines connecting nodes reflect the strength of cooperation. **(B)** An overlay visualization of the years for national (regional) publications. Colors turning towards yellow represent time getting closer.

In total, 64 countries were involved in the research of glucose metabolism reprogramming in BC, including 34 countries each with a minimum of 5 publications. China had the most publications (n=331), followed by the United States of America (USA) (n=330), the United Kingdom (UK) (n=68), India (n=52), and Italy (n=51) (**Table 1**). Although China was comparable to the USA in publication output, its number of citations was only half of that in the USA, indicating that the USA had a greater academic influence in this research field. A visualization analysis of cooperation among countries (regions) indicated a close partnership between China and the USA in the research of glucose metabolism reprogramming in BC (**Figure 3A**). The temporal distribution of publications by countries visualized that the USA was a pioneer to drive the research of glucose metabolism reprogramming in BC, followed by Japan, Germany, and China (**Figure 3B**). Research in this field started late in India and Turkey.

**Table 1:** The top 10 countries (regions) contributing to the publications of glucose metabolism reprogramming in BC.

Rank	Country (region)	Publications	Citations	TLS
1	China	331	13,620	84
2	USA	330	27,875	216
3	UK	68	7,472	76
4	India	52	1,186	28
5	Italy	51	3,765	40
6	Germany	38	1,232	30
7	Canada	32	3,113	33
8	South Korea	30	1,369	14
9	Japan	27	1,072	32
10	Spain	24	1,681	17

BC, breast cancer; TLS, total link strength; USA, the United States of America; UK, the United ingdom.

### 3.3 Distribution of institutions

**Figure 4:**
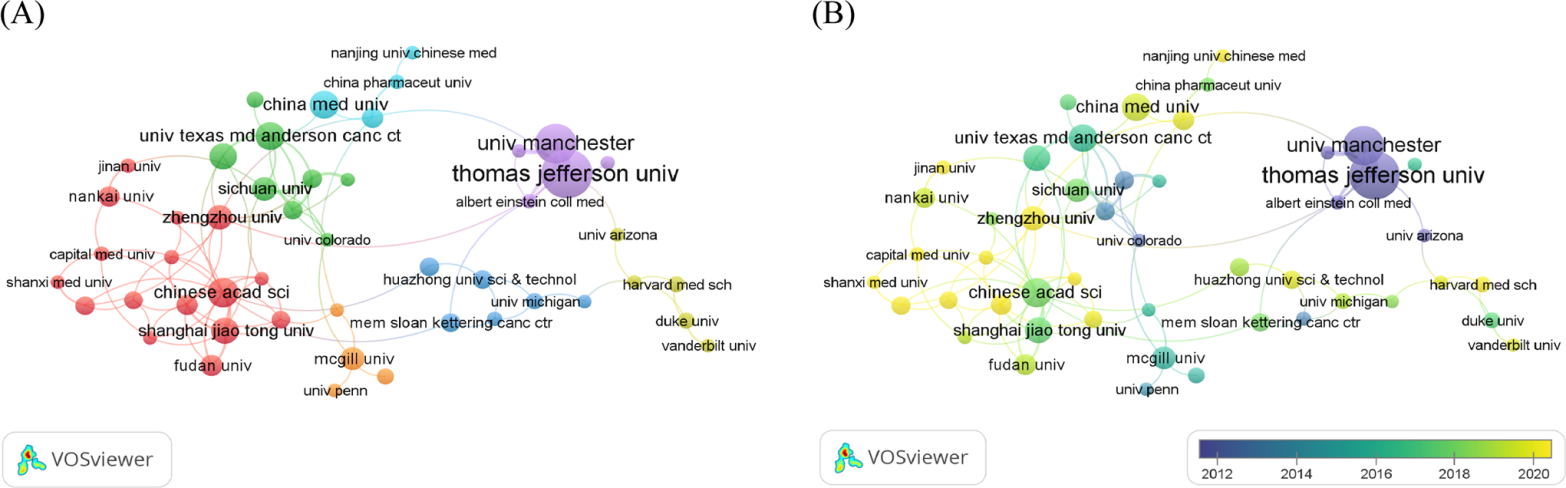
Institutional distribution of publications of glucose metabolism reprogramming in BC. **(A)** A network visualization of institutions contributing to the publication outputs, and their corporation. The sizes of nodes reflect the number of publications by institutions, and the thicknesses of the lines connecting nodes reflect the strength of cooperation. **(B)** An overlay visualization of the year for publications in global institutions. Colors turning towards yellow represent time getting closer.

A total of 1,358 institutions worldwide participated in the research of glucose metabolism reprogramming in BC, most of which were universities. Among the 79 institutions with more than 5 publications, Thomas Jefferson University ranked first in the number of publications, followed by the University of Manchester, the Chinese Academy of Sciences, China Medical University, and the University of Texas MD Anderson Cancer Center (**Table 2**). Notably, Thomas Jefferson University in the USA ranked first in both the publication and citation counts, as well as the overall strength of corporation, indicating its profound contribution in this research field. An institutional cooperation is shown in **Figure 4A**, with more collaborations intersected within the same country. The largest cluster highlighted in red consisted of 16 institutions mainly from China. Temporal dynamics visualized that international cooperations, such as that between Thomas Jefferson University in the USA and the University of Manchester in the UK, contributed greatly to the early research of glucose metabolism reprogramming in BC (**Figure 4B**). From 2018, a new torrent in this research field has been waged by Chinese institutions.

**Table 2:** The top 10 institutions contributing to the publication outputs of glucose metabolism reprogramming in BC.

Rank	Institution	Country (region)	Publications	Citations	TLS
1	Thomas Jefferson University	USA	40	6,497	43
2	University of Manchester	UK	30	4,574	36
3	Chinese Academy of Sciences	China	19	1,640	28
4	China Medical University	China	18	567	2
5	The University of Texas M.D. Anderson Cancer Center	USA	18	1,682	12
6	Shanghai Jiao Tong University	China	16	1,181	17
7	Sun Yat-sen University	China	16	1,705	6
8	Zhengzhou University	China	15	263	11
9	Sichuan University	China	14	1,452	12
10	McGill University	Canada	13	2,158	4

BC, breast cancer; TLS, total link strength; USA, the United States of America; UK, the United Kingdom.

### 3.4 Distributions of authors and co-cited authors

**Figure 5:**
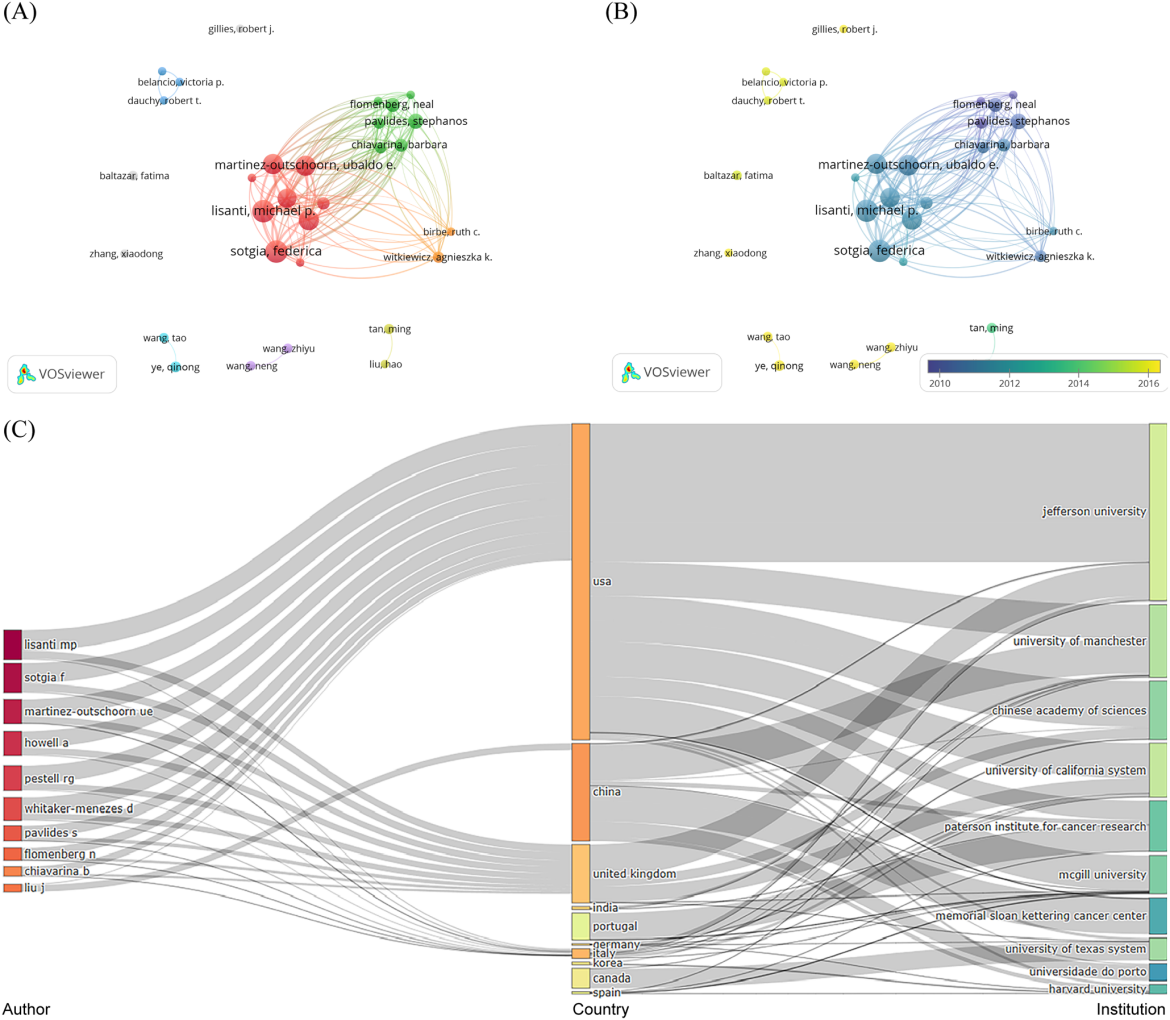
Distribution of authors and co-cited authors in the research field of glucose metabolism reprogramming in BC. **(A)** A network of authors’ collaborations. The sizes of nodes reflect the numbers of publications issued by authors, and the thicknesses of the lines connecting nodes reflect the strength of author collaborations. **(B)** An overlay visualization of the years for publications issued by authors. Colors turning towards yellow represent time getting closer. **(C)** A three-field plot visualizing the authors, countries (regions), and institutions.

A total of 5,839 authors were engaged in the publications of glucose metabolism reprogramming in BC, involving 30 authors having published more than five articles. The top three authors with the most publications were Lisanti, Michael P. (n=33), Sotgia, Federica (n=33), and Martinez-Outschoorn, Ubaldo E. (n=28) (**Table 3**), all of whom ranked at the top in citation counts. The three-field plot visualized the network of the top 10 authors from the corresponding countries and institutions with the largest contribution to the publication outputs (**Figure 5C**). Although Frank, Philippe G. only published five articles, his average citation count was the highest, at 398.4 times, suggesting his concrete impact on the research field of glucose metabolism reprogramming in BC. As shown in **Figure 5A**, author cooperations were mainly clustered into seven groups, and those highlighted in red, green, and orange indicated a closer collaboration. Authors who frequently cooperated with others were mainly from Thomas Jefferson University in the USA and the University of Manchester in the UK. The overlay visualization map of the years of publications by clustered authors indicated an accelerating pace of Chinese researchers in investigating glucose metabolism reprogramming of BC in recent years (**Figure 5B**). Nevertheless, cooperation between different research groups seemed not to be frequent, indicating that there is still room for further development in building a closer academic cooperation network. A total of 34,614 co-cited authors participated in the research of glucose metabolism reprogramming in BC, and the top three were Warburg Otto (n=540), Martinez-Outschoorn, Ubaldo E. (n=314), and Heiden, MGV (n=313). The high citation frequency of co-cited authors directly reflected their foundational contributions.

**Table 3:** The top 10 authors with high publication outputs and co-citations.

Rank	Author	Publications	Citations	H-index	Author	Co-citations
1	Lisanti, Michael P.	33	5,996	32	Warburg Otto	540
2	Sotgia, Federica	33	5,996	32	Martinez-Outschoorn, Ubaldo E.	314
3	Martinez-Outschoorn, Ubaldo E.	28	5,227	28	Heiden, MGV	313
4	Howell, Anthony	27	4,330	27	DeBerardinis, R. J.	299
5	Pestell, Richard G.	27	5,266	26	Hanahan, Douglas	293
6	Whitaker-Menezes, Diana	25	5,011	25	Semenza, GL	272
7	Pavlides, Stephanos	16	3,934	16	Gatenby, RA	217
8	Flomenberg, Neal	13	3,536	13	Pavlides, Stephanos	212
9	Chiavarina, Barbara	11	2,049	11	Dang, CV	187
10	Lin, Zhao	10	1,709	10	Kim, JW	143

### 3.5 Distributions of journals and co-cited journals

**Figure 6:**
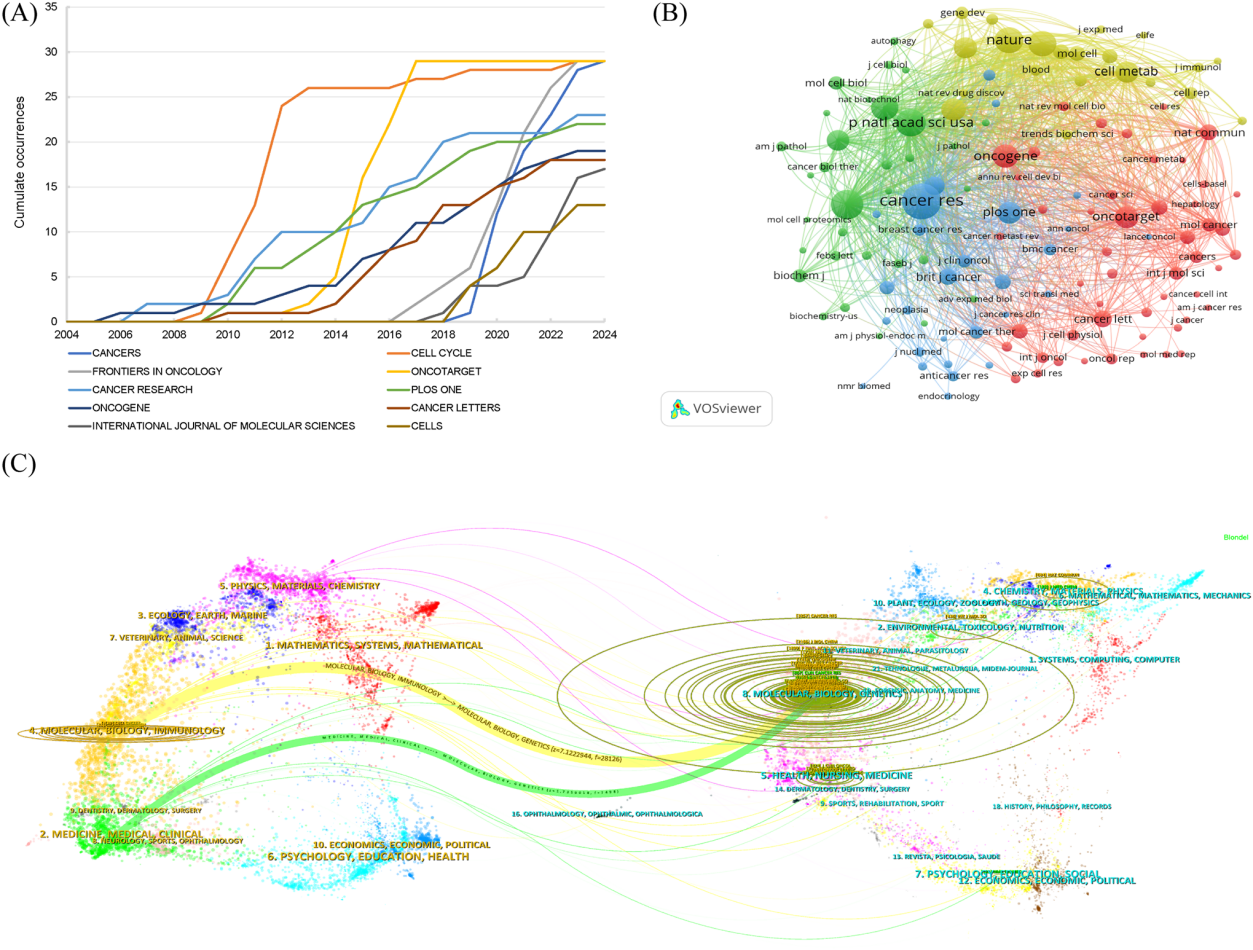
Distribution of journals and co-cited journals in the research field of glucose metabolism reprogramming in BC. **(A)** Annual publication outputs of the top 10 journals. **(B)** A network visualization of co-cited journals. The sizes of nodes reflect the number of journal co-citations, and the thicknesses of the lines connecting nodes reflect the strength of co-cited occurrence between journals. **(C)** A dual-map overlay visualization of journals, with the disciplines on the left covered by the citing journals and those on the right of the cited journals.

Articles reporting the glucose metabolism reprogramming in BC have been published in 377 journals. The top 10 journals ranked by publication outputs are listed in **Table 4**, with 23.82% of articles published in these journals. There showed an annually increasing trend in the publications (**Figure 6A**). Notably, *Cancers*, *Cell Cycle*, *Frontiers in Oncology*, and *Oncotarget* each had 29 publications, and *Cell Cycle* was equipped with the highest number of citations (n=5,043). Among the 4,212 co-cited journals, 132 had been cited for more than 100 times. Distribution of co-cited journals is visualized in **Figure 6B**. The top three journals ranked by co-citation frequency were *Cancer Research*, *Journal of Biological Chemistry*, and *Proceedings of the National Academy of Sciences of the United States of America*. The dual-map overlay of journals depicted two citation pathways, with the disciplines on the left (Molecular, Biology, Immunology and Medicine, Medical, Clinical) covered by the citing journals and those on the right (Molecular, Biology, Genetics) of the cited journals (**Figure 6C**). It reflected an in-depth interaction and knowledge flow in interdisciplinary research.

**Table 4:** The top 10 journals with high publication outputs and co-citations.

Rank	Journal	Publications	IF (2023)	JCR (2023)	Journal	Co-citations	IF (2023)	JCR (2023)
1	Cancers	29	4.5	Q1	Cancer Research	3,257	12.5	Q1
2	Cell Cycle	29	3.4	Q3	Journal Of Biological Chemistry	2,170	4	Q2
3	Frontiers in Oncology	29	3.5	Q2	Proceedings Of The National Academy Of Sciences Of The United States Of America	1,899	9.4	Q1
4	Oncotarget	29	–	–	Nature	1,737	50.5	Q1
5	Cancer Research	23	12.5	Q1	Cell	1,627	45.5	Q1
6	PLOS One	22	2.9	Q1	Science	1,572	44.7	Q1
7	Oncogene	19	6.9	Q1	Oncogene	1,420	6.9	Q1
8	Cancer Letters	18	9.1	Q1	Nature Reviews Cancer	1,288	72.5	Q1
9	International Journal of Molecular Sciences	17	4.9	Q2	Oncotarget	1,203	–	–
10	Cells	13	5.1	Q2	PLOS one	1,201	2.9	Q1

IF, impact factor; JCR, journal citation reports.

### 3.6 Distribution of citations

A total of 48,452 references have been cited in 957 publications, involving 73 references co-cited for more than 30 times. The top 10 most co-cited references are listed in **Table 5**. They generally focused on the description of metabolic reprogramming characteristics of cancer cells, including key metabolic pathways, regulatory factors, and therapeutic strategies. In 2009, Heiden, MGV et al. published an article entitled *Understanding the Warburg effect: the metabolic requirements of cell proliferation in the journal of Science*, serving as the most frequently cited reference (n=293). It summarized the metabolic demands for cell proliferation pointing out that oncogenic mutations can lead to an increased uptake of nutrients (especially glucose), and demonstrated the therapeutic potential of metabolic dependencies in the anti-cancer treatment. Through analyzing the publication dynamics, the Warburg effect has been re-recognized for its critical role in the complex relationship between cellular metabolism and proliferation.

**Table 5:** The top 10 co-cited references.

Rank	Title	First Author	Journal	Co-citations	Year
1	Understanding the Warburg effect: the metabolic requirements of cell proliferation	Heiden, MGV	Science	293	2009
2	On the origin of cancer cells	Warburg Otto	Science	263	1956
3	Hallmarks of cancer: the next generation	Hanahan, Douglas	Cell	238	2011
4	Why do cancers have high aerobic glycolysis?	Gatenby, RA	Nature Reviews Cancer	146	2004
5	The biology of cancer: metabolic reprogramming fuels cell growth and proliferation	DeBerardinis, RJ	Cell Metabolism	111	2008
6	The reverse Warburg effect: aerobic glycolysis in cancer associated fibroblasts and the tumor stroma	Pavlides, Stephanos	Cell Cycle	102	2009
7	Regulation of cancer cell metabolism	Cairns, RA	Nature Reviews Cancer	101	2011
8	The M2 splice isoform of pyruvate kinase is important for cancer metabolism and tumour growth	Christofk, HR	Nature	96	2008
9	The metabolism of tumors in the body	Warburg Otto	Journal of General Physiology	95	1927
10	Aerobic glycolysis: meeting the metabolic requirements of cell proliferation	Lunt, SY	Annual Review of Cell and Developmental Biology	93	2011

### 3.7 Distribution of most-cited publications

Among the 957 publications reporting the glucose metabolism reprogramming of BC, 123 have been cited for more than 100 times. The top 10 most-cited publications are listed in **Table 6**. *From Krebs to clinic: glutamine metabolism to cancer therapy*, published by Altman, Brian J. et al. in Nature Reviews Cancer was the most frequently cited publication. In this review, cancer cells are found to promote the tricarboxylic acid cycle to participate in the biosynthesis of nucleotides and fatty acids in a glutamine-dependent way when they are unable to effectively utilize glucose. Moreover, nutrients to fuel their metabolism vary by types of BC cell lines. The second most-cited article published by Pavlides, Stephanos et al. in 2009 proposed that the reverse Warburg effect in cancer-associated fibroblasts (CAFs), which are the most abundant type of cells in the tumor microenvironment (TME), is a distinguished characteristic from the Warburg effect in cancer cells. They found that lactate and pyruvate secreted by CAFs through aerobic glycolysis are absorbed and utilized by nearby cancer cells, and verified this mechanism in matrix cells deficient in caveolin-1 (Cav-1). Loss of Cav-1 in human BC stromal cells, particularly, is associated with tumor recurrence, metastasis, and poor prognosis.

**Table 6:** The top 10 most-cited publications.

Rank	Title	First Author	Citations	Year
1	From Krebs to clinic: glutamine metabolism to cancer therapy	Altman, Brian J.	1,217	2016
2	The reverse Warburg effect: Aerobic glycolysis in cancer associated fibroblasts and the tumor stroma	Pavlides, Stephanos	1,019	2009
3	Targeting cellular metabolism to improve cancer therapeutics	Zhao, Y.	786	2013
4	The role of disturbed pH dynamics and the Na^+^/H^+^ exchanger in metastasis	Cardone RA	723	2005
5	Mitochondrial mutations in cancer	Brandon, M.	630	2006
6	SIRT3 opposes reprogramming of cancer cell metabolism through HIF1α destabilization	Finley, Lydia W. S.	625	2011
7	Hexokinase 2 is required for tumor initiation and maintenance and its systemic deletion is therapeutic in mouse models of cancer	Patra, Krushna C.	610	2013
8	Lactate influx through the endothelial cell monocarboxylate transporter MCT1 supports an NF-κB/IL-8 pathway that drives tumor angiogenesis	Vegran, Frederique	576	2011
9	Disrupting proton dynamics and energy metabolism for cancer therapy	Parks, Scott K.	471	2013
10	Ketones and lactate “fuel” tumor growth and metastasis	Bonuccelli, Gloria	465	2010

### 3.8 Keyword analyses

#### 3.8.1 Co-occurrence of keywords

**Figure 7:**
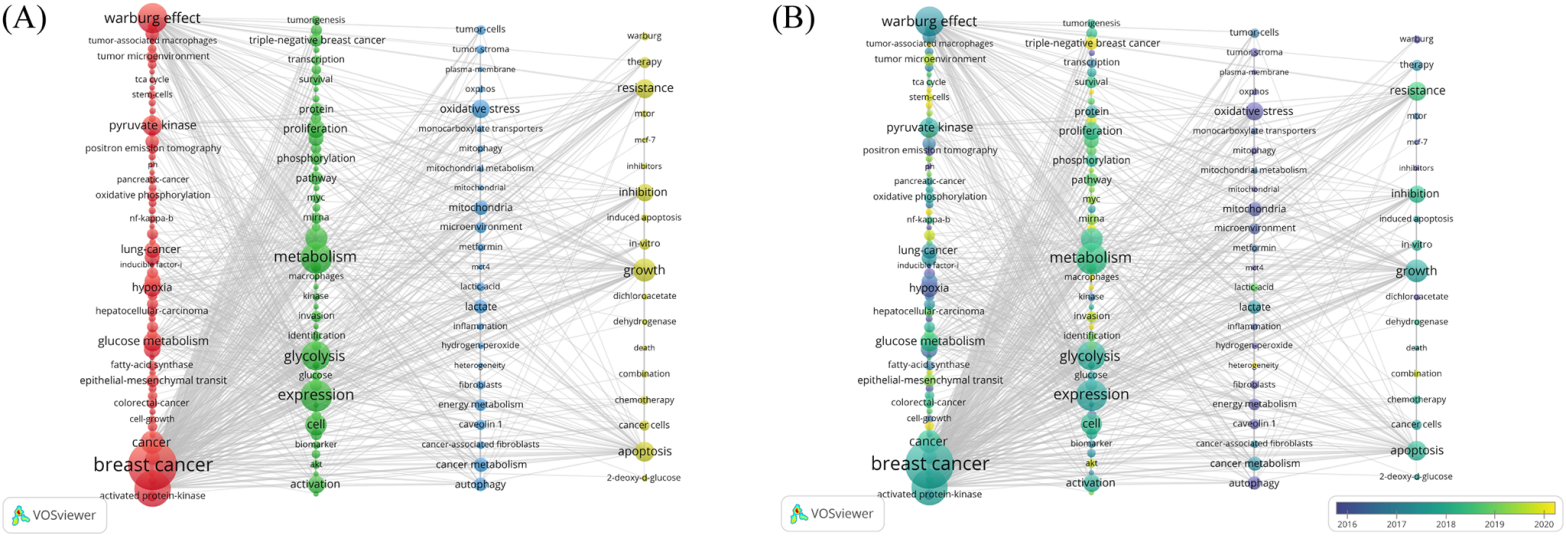
Clustering of keywords. **(A)** Cluster visualization of keywords. The sizes of nodes reflect the frequency of keywords, and the thicknesses of the lines connecting nodes reflect the strength of keyword co-occurrence. **(B)** An overlay visualization of the time of keyword occurrence. Colors turning towards yellow represent time getting closer.

The co-occurrence analysis of keywords can reveal the distribution of hot topics within the research field. A total of 4,106 keywords were initially identified by merging synonyms in VOSviewer, and 154 keywords having appeared for more than 10 times were finally obtained, after removing those having frequent occurrences but lacking analytical significance. They were clustered into four groups, including BC metabolism and Warburg effect, glucose metabolism and molecular biology of BC cells, glucose metabolism and the TME in BC, and glucose metabolism and therapeutic strategies in BC, as shown from left to right in **Figure 7A**. A temporal distribution of the above keywords is visualized in **Figure 7B**. Notably, TME-based tumor stroma was analyzed in earlier research, and as the color changes in the figure, the research gradually deepened, and the relationship between tumor metabolic transformation and the entire TME became increasingly prominent. Targeted therapy, immunotherapy, lncRNA, and triple-negative breast cancer (TNBC) have appeared later as keywords, indicating that the burgeoning research has highlighted therapeutic strategies, especially for TNBC with a worse prognosis in BC.

#### 3.8.2 Keyword bursts

**Figure 8:**
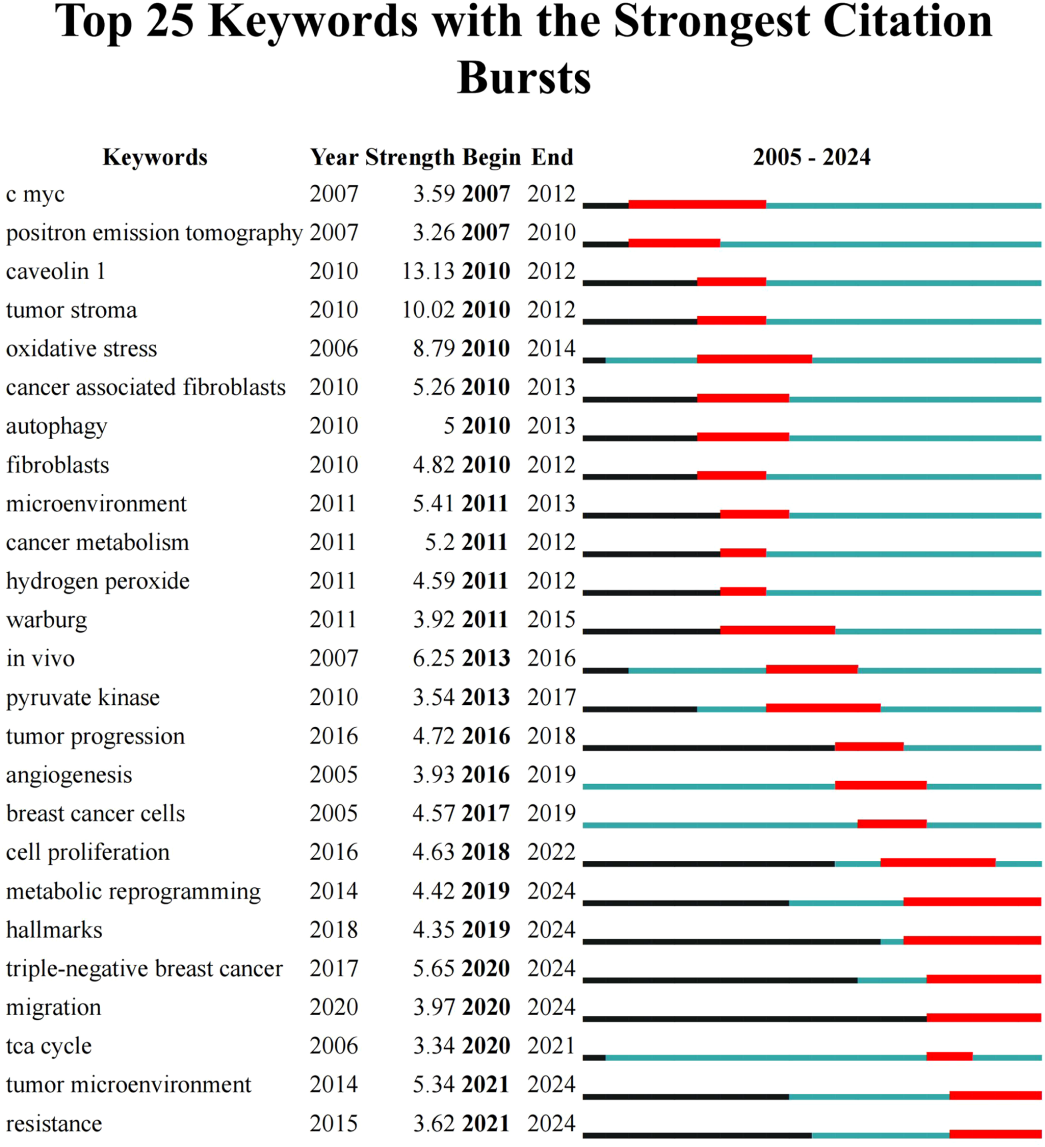
The top 25 keywords with the strongest citation bursts. The term “Year” indicates the initial emergence of the keyword; “Begin” and “End” mark the starting and concluding years of the keyword’s status as cutting-edge, respectively; ‘Strength’ reflects the emergent strength.

Keyword burst analysis aims to unveil the frequency of keyword occurrences in publications over a period of time, thus showing the academic importance and changing trends of the keywords within the field. As shown in **Figure 8**, the top 25 keywords with the strongest citation bursts were presented, with caveolin 1 (13.13) ranking first, followed by tumor stroma (10.02). Keywords presented a strong burst within the past three years included metabolic reprogramming (2019–2024), hallmarks (2019–2024), TNBC (2020–2024), migration (2020–2024), TME (2021–2024), and resistance (2021–2024), indicating the latest research hotspots.

## 4 Discussion

### 4.1 Overview of the research on glucose metabolism reprogramming in BC

Global publication outputs associated with glucose metabolism reprogramming in BC have shown an overall increasing trend over the past two decades. Since the first publication in 2005, an explosive growth in annual publication outputs in 2010 and 2015 was largely attributed to the great strides on medical techniques detecting cell proliferation and metabolomics. Furthermore, the rising global burden of BC, combined with the recognition of “Deregulating cellular energetics” as a distinctive feature of cancer (14), has led to a deeper exploration into glucose metabolism reprogramming in BC.

China and the USA contributed the highest number of publications in this field, while the USA boasted of the highest citation frequency. The temporal analysis showed earlier publications from the USA than those from China, indicating that the USA has pioneered in the research of glucose metabolism reprogramming in BC. Notably, China has caught up from behind in recent years as evidenced by its leadership in the number of institutions and output of publications in this field. In addition, our bibliometric analysis uncovered close domestic collaborations, and to-be-expanded international collaborations.

The top three authors with the highest publication outputs have closely collaborated to explore novel therapeutic strategies for BC via acting on the epithelial-stromal metabolic coupling, particularly the reverse Warburg effect. They co-authored an article titled Cancer metabolism: a therapeutic perspective, published in *Nature Reviews Clinical Oncology* in 2017. They highlighted the superior therapeutic potential of modulating cancer metabolism, rather than conventional genetic mutations. Recently, metabolic characteristics of cancer stem cells (CSCs), acknowledged as the origin of tumor recurrence and drug resistance, have slipped into the spotlight of research. Through screening BC cells with CSC characteristics using the SRY-box 2 (SOX2) transcription factor, they illustrated metabolic overactivity in MCF-7 cells overexpressing SOX2, manifesting as enhanced glycolysis rate, and Tamoxifen resistance (15). Considering the high heterogeneity among metabolic phenotypes of CSCs, future research is required to clearly state the complex mechanisms underlying the maintenance of stemness and metabolic reprogramming in CSCs of BC (16).

The distribution of journals and co-cited journals reflected the high quality of publications, as 50% of the top 10 journals ranked by publication outputs could be classified as Q1 and Q2 by the Journal Citation Reports (JCR). Although the journal *Nature Reviews Cancer* does not stand in the top 10, it has published three highly cited articles among the top 10 most-cited publications. Both the outputs in one journal and the quality and impact of the articles it publishes should be concerned during the bibliometric analysis of journal metrics. The dual-map overlay of journals demonstrated the trajectory between citing and cited journals, illustrating the research change from fundamental molecular biology to clinical applications, as well as highlighting the importance of translational medicine.

### 4.2 Research hotspots and future trends of glucose metabolism reprogramming in BC

Co-occurrence and burst analyses of keywords were performed to unveil current hot topics and future trends in reprogramming glucose metabolism of BC.

Therapeutic strategies for BC differ from immunohistochemical biomarkers categorized as Luminal A, Luminal B, HER2-positive, and triple-negative subtypes (17). Classified by the molecular subtypes of BC, Luminal A/B subtype show a tendency towards oxidative phosphorylation (OXPHOS), whereas TNBC exhibits a preference to glycolysis with high invasiveness and low oxygen consumption (18). Therefore, glucose metabolism reprogramming is believed to profoundly affect the biological characteristics of malignancies. In the present study, the keyword TNBC occurred later but served as a hot topic in recent years. Despite the low incidence of TNBC among other types of BC, it lacks effective therapeutic targets (19). Glucose metabolism reprogramming may be targeted to design promising strategies against TNBC. Previous evidence has shown that the upregulation of glycolysis-related enzymes (e.g., hexokinase, pyruvate kinase, and phosphofructokinase) and transport proteins (e.g., glucose transporters and monocarboxylate transporters) in TNBC is associated with the activation of certain signaling pathways and transcription factors (20). The role of non-coding RNAs in the Warburg effect of BC has been recently clarified (21). Du et al. found that miR-210-3p is upregulated in TNBC to promote aerobic glycolysis by mediating activities of HIF-1α and p53 (22). Glycolysis aggravates the malignancy of TNBC, offering new avenues for exploring metabolism-targeted therapeutic strategies (23). In fact, the metabolic phenotype of TNBC cells cunningly switches between glycolysis and OXPHOS to resist against targeted drugs. However, further research is needed to elucidate how breast cancer cells regulate this hybrid metabolic state at the genetic level (24). Existing studies have found that the combination of two antimetabolic drugs, metformin and 2-deoxy-D-glucose (2-DG), can synergistically act on this hybrid metabolic state, not only inhibiting the growth and metastasis of BC cells but also holding promise as an adjuvant therapy for TNBC immunotherapy. Targeting both glycolysis and OXPHOS is therefore considered as a novel direction of future research (25).

Burst analysis of keywords pointed out TME as a continuous hot topic in the glucose metabolism reprogramming of BC. The TME is closely linked with glucose metabolism of BC, and its variation disrupts nutrient supply to immune and stromal cells, and leads to issues such as tumor immune escape (26–28). Cav-1 in human BC stromal cells, with the strongest burst strength, was greatly attributed to the deep mining of the reverse Warburg effect of CAFs (29). A hypoxic environment induces CAFs into a metabolic supporter of BC cells (30). However, it is still challenging to fully explain the complex mechanisms of glucose metabolism reprogramming between the tumor and stromal cells by the reverse Warburg effect alone. A new study found that CAFs could form contact-dependent tunneling nanotubes with BC cells for material exchange, including mitochondrial transfer, which could influence tumor metabolism and facilitate migration (31). In addition, immune cells in the TME such as tumour-associated macrophages (TAMs) are widely recruited in aggressive BC subtypes (32). Furthermore, the metabolism of the tumour is able to induce their polarization into different functional phenotypes (33). Jiang et al. found that lactate produced by aerobic glycolysis in BC cells promotes the polarization of TAMs into the M2 type that favors tumor growth (5). Conversely, TAMs also enhance glycolysis in BC cells by delivering HIF-1α-stabilizing long non-coding RNA (HISLA) via the release of extracellular vesicles, thus allowing a better adaptation to TME. For this reason, aptamer–siRNA chimeras can be developed to silence HISLA in TAMs (34).

Resistance was an emerging keyword with a strong burst strength. Drug resistance seriously challenges the treatment of BC. Estrogen receptor-positive (ER+) BC is the predominant subtype of BC that initially responds well to endocrine therapy, but eventually develops drug resistance (35). Previous studies have mainly explored the involvement of ER signal transduction in the resistance to endocrine therapy, but underestimated the glucose metabolism reprogramming (36–38). Building on previous research, Lisanti, Michael P. et al. discovered that fibroblasts, when co-cultured with MCF-7 cells, had the ability to induce Tamoxifen-resistance in ER-positive BC cells. Moreover, they observed that the co-treatment of Dasatinib with Tamoxifen caused a rapid change in the metabolic phenotype of the MCF-7 cells, thereby overcoming this resistance. In this process, Dasatinib was found to prevent the loss of stromal Cav-1. The authors thus suggested that the Warburg effect may offer a therapeutic solution for drug resistance (39). Their research team has recently discovered that overexpression of transcription factor FoxO3a in BC cells restores the response of resistant cells to Tamoxifen via mediating the energy metabolism pathways (40). Song’s group mentioned above, again from the lncRNA perspective, reported that overexpression of lncRNA DIO3OS enhances aerobic glycolysis and causes resistance to aromatase inhibitors, reflecting the potential role of lncRNAs as therapeutic targets for modulating key enzymes of glycolytic metabolism in response to endocrine therapy (41). Drug resistance is a permanent issue bothering the treatment of almost all subtypes of BC (42). A combination of conventional anti-cancer drugs and glycolytic inhibitors is expected to overcome drug resistance and benefit BC patients (43).

Preclinical studies have demonstrated promising anti-metabolic capabilities of innovative drugs, although they have not been successfully translated into clinical practice yet (44, 45). Further verification is required to confirm the anti-metabolic effects of glycolytic inhibitors, either as a monotherapy or combined therapy (46). Lonidamine (LND) is known to target glucose metabolism, but has a limited efficacy when applied alone. It can be clinically utilized as a chemosensitizer (47). Doxorubicin (DOX), a common chemotherapeutic agent for BC, decreases glucose uptake via downregulating glucose transporter-1 and hexokinase II (48). In advanced BC patients with liver metastases, DOX combined with LND provide a significantly higher response rate than the DOX monotherapy (49). We therefore suggested that future research should focus on the combination treatment that exert maximal therapeutic efficacy and minimal toxicity by targeting glucose metabolism reprogramming of BC. As a glucose analog, 2-deoxy-D-glucose (2-DG) has been widely recognized for its diagnostic value in tumor imaging when conjugated with radioactive isotopes. It accumulates significantly within cells by competing with glucose for entry under hypoxic conditions, which further disrupts the glycolytic process and thereby exerts cytotoxic effects. However, due to its poor drug-like properties, 2-DG is often used in combination with other antitumor drugs (50). Researchers have found that modified 2-DG can enter cells through passive diffusion without reliance on glucose transport proteins, while also improving therapeutic effects by increasing its half-life and oral bioavailability (51). In the future, novel 2-DG derivatives can be developed to enhance the value of 2-DG in BC treatment. The non-toxic treatment strategy represented by the ketogenic diets has garnered attention in the treatment of BC. Limiting the supply of carbohydrates can shift glucose metabolism towards lipid metabolism, thereby blocking the Warburg effect. Studies have shown that ketone bodies can inhibit the growth and proliferation of various cancer cells, including BC, without affecting normal cells. This is because normal cells can utilize acetyl CoA from ketone bodies and produce ATP through the Krebs cycle, while cancer cells cannot, thus achieving a strong inhibition of glycolysis (52). Due to the selective effect of ketone bodies on cancer cell proliferation, they have also been found to reduce the IC50 values of chemotherapeutic agents in treating cancers. Miller et al. found that the IC50 value was reduced by a factor of almost 5 when ketone bodies were used in combination with rapamycin in MCF-7 cells (53), and the two showed a strong synergistic effect in the treatment of BC *in vivo* (54). This provides us with a new perspective for exploring combined treatment strategies for BC.

## 5 Limitations

Limitations present in the present bibliometric analysis. First, we only analyzed English-language publications from the WOSCC database, leading to potential biases. Second, bibliometric analysis of citation counts may result in an underestimation of citations in newly published articles.

## 6 Conclusion

The present bibliometric analysis provides the research progresses and hotspots on the glucose metabolism reprogramming of BC over the past two decades. Globally, academic institutions from various countries and regions have been engaged in an extensive exploration of the glucose metabolism reprogramming in BC. China and the USA have particularly dominated the scientific research in this field. In recent years, therapeutic strategies for BC, especially a combination therapy, by targeting glucose metabolism reprogramming and preventing drug resistance, have gradually replaced mechanism-oriented research, but there still lack clinical trials to assess their efficacy and safety. High-quality studies are essential in the future to provide survival benefits to BC patients.

## Data Availability

The original contributions presented in the study are included in the article/supplementary material. Further inquiries can be directed to the corresponding author.
